# Supplementary material for: Open-source, high performance miniature 2-photon microscopy systems for freely behaving animals
Source: Nat Commun. 2025 Aug 3;16:7125. doi: 10.1038/s41467-025-62534-y (PMC12318034; doi:10.1038/s41467-025-62534-y)
Supplement: Supplementary file 1 — Supplementary Information [file 41467_2025_62534_MOESM1_ESM.pdf]

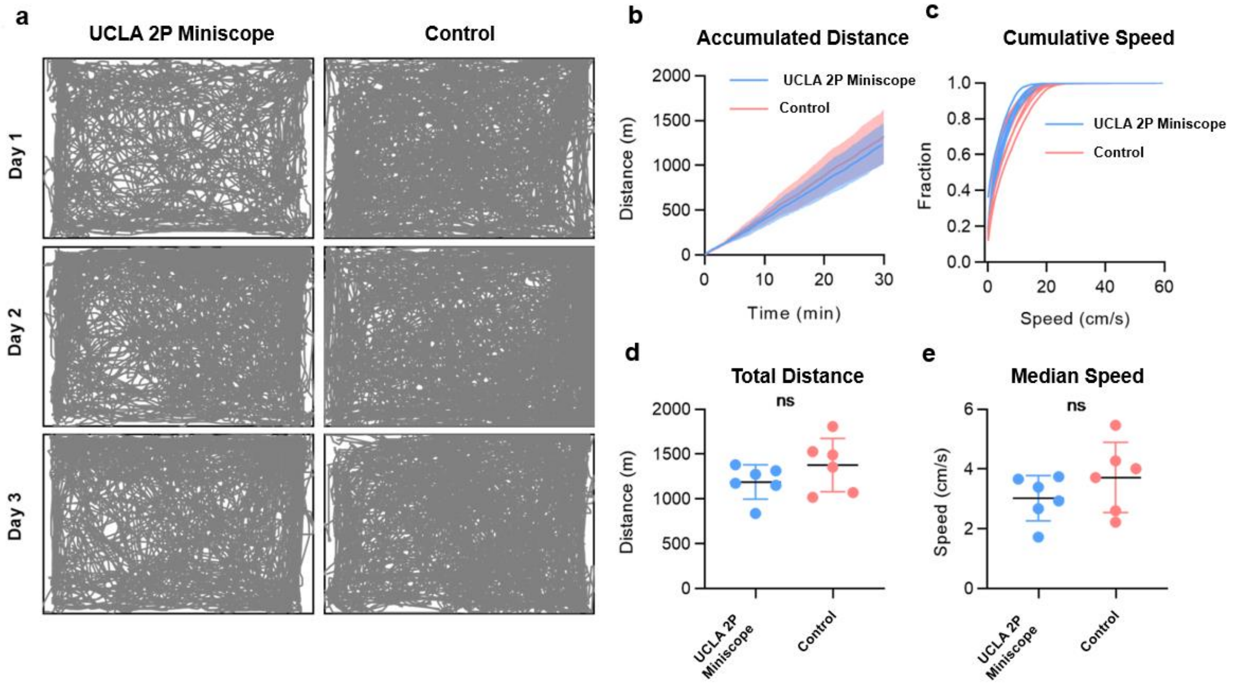

Supplementary Figure 1: UCLA 2P Miniscope does not interfere with free-foraging behavior in mice. a) Three days representative trajectories of one mouse running in an approximately 38cm x 28cm open field, with 2 trials per day: UCLA 2P Miniscope with cable assembly, control (no head mounted device or cabling). b) Accumulated distance over 30 min of running. Lines, mean across 2 mice (each animal includes 3 days trajectories). Shaded region, SD at all time points. Color indicates experimental condition. Note the similarity between UCLA 2P Miniscope and control group. c) Cumulative speed distribution over 30 min of running. Each curve shows one trial. Color indicates experimental condition. d) Scatter plot showing total travel distance in each condition (30 min each). Horizontal lines indicate mean; Error bars indicate SD. Colored dots, individual trials. Conditions are compared using paired t-test, ns $p>0.05$. e) Scatter plot showing median speed in each condition (30 min each). Horizontal lines indicate mean; Error bars indicate SD. Colored dots, individual trials. Conditions are compared using paired t-test, ns $p>0.05$.

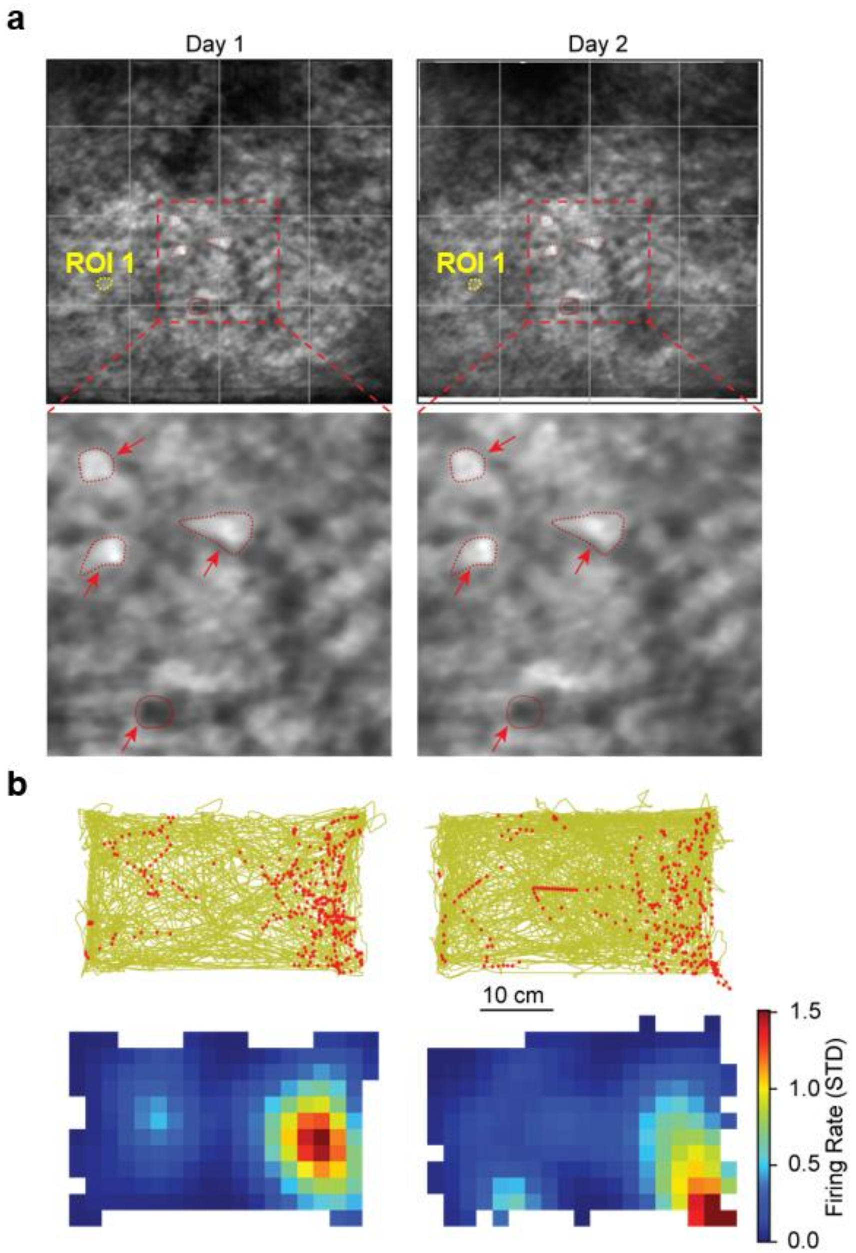

Supplementary Figure 2: UCLA 2P Miniscope enables multi-day tracking of the same cells in DG. a) The same FOV from DG across two days. Red dashed squares show the zoom-in areas (bottom) with red arrows highlighting the same features. Images are the mean of registered frames from Suite2P [4]. Day 2 image is calibrated using CellReg [5]. b) Firing fields for a representative cell (yellow circles labeled ROI 1 in panel a) detected across two days. (Top) Trajectories with spike locations; (Bottom) corresponding color-coded rate maps.

a

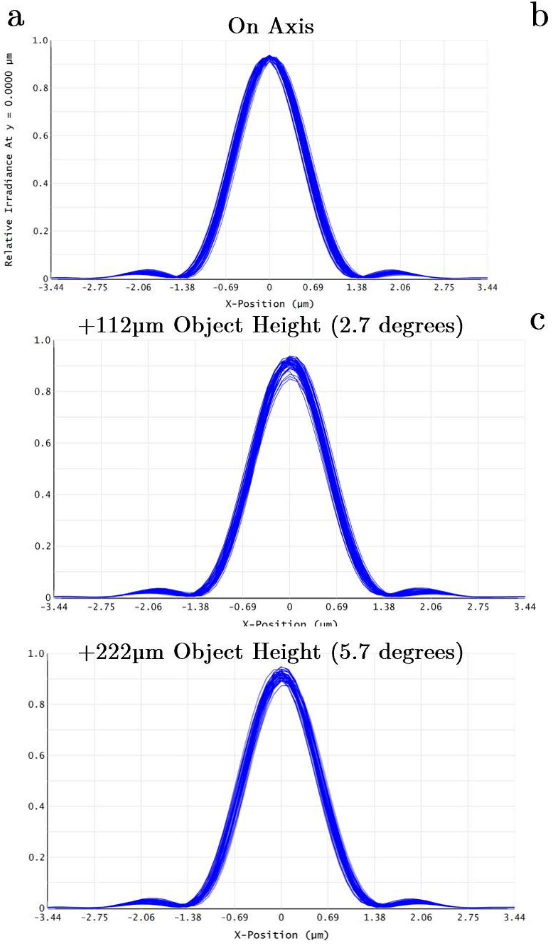

b

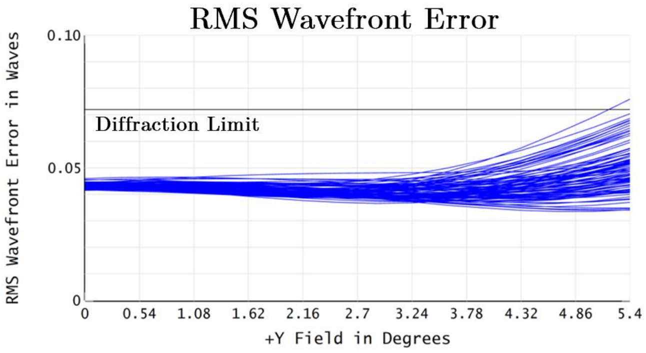

c

	Type	Surf	Code	Nominal	Min	Max	Comment
1	COMP	19	0	0.223	-0.050	0.050	Default compensator on back focus.
2	TWAV				0.920		Default test wavelength.
3	TTHI	2	5	4.000	-0.050	0.050	Scan Lens 1 Mounting Thickness
4	TTHI	3	5	2.200	-2.500E-03	2.500E-03	Scan Lens 1 Element 1 Thickness Tolerance
5	TTHI	4	5	1.600	-2.500E-03	2.500E-03	Scan Lens 1 Element 2 Thickness Tolerance
6	TTHI	5	8	1.750	-0.050	0.050	Scan Len 2 Mounting Thickness
7	TTHI	6	8	2.200	-2.500E-03	2.500E-03	Scan Lens 2 Element 1 Thickness Tolerance
8	TTHI	7	8	1.600	-2.500E-03	2.500E-03	Scan Lens 2 Element 2 Thickness Tolerance
9	TTHI	8	11	9.570	-0.050	0.050	Tube Lens Mounting Thickness
10	TTHI	9	11	1.000	-2.500E-03	2.500E-03	Tube Lens Element 1 Thickness Tolerance
11	TTHI	10	11	3.400	-2.500E-03	2.500E-03	Tube Lens Element 2 Thickness Tolerance
12	TTHI	11	12	2.000	-0.050	0.050	Objective Mounting Thickness
13	TTHI	12	14	2.500	-2.500E-03	2.500E-03	Objective Lens Element 1 Thickness Tolerance
14	TTHI	13	0	0.500	-2.500E-03	2.500E-03	Objective Lens Element 2 Thickness Tolerance
15	TTHI	16	14	1.300	-2.500E-03	2.500E-03	Objective Lens Element 3 Thickness Tolerance
16	TEDX	3	5	0.000	-0.075	0.075	Scan Lens 1 Decenter (X)
17	TEDY	3	5	0.000	-0.075	0.075	Scan Lens 1 Decenter (Y)
18	TEDX	6	8	0.000	-0.075	0.075	Scan Lens 2 Decenter (X)
19	TEDY	6	8	0.000	-0.075	0.075	Scan Lens 2 Decenter (Y)
20	TEDX	9	11	0.000	-0.075	0.075	Tube Lens Decenter (X)
21	TEDY	9	11	0.000	-0.075	0.075	Tube Lens Decenter (Y)
22	TEDX	12	19	0.000	-0.050	0.050	Objective Assembly Decenter (X)
23	TEDY	12	19	0.000	-0.050	0.050	Objective Assembly Decenter (Y)
24	TETX	3	5	0.000	-0.250	0.250	Scan Lens 1 Tilt (X)
25	TETY	3	5	0.000	-0.250	0.250	Scan Lens 1 Tilt (Y)
26	TETX	6	8	0.000	-0.250	0.250	Scan Lens 2 Tilt (X)
27	TETY	6	8	0.000	-0.250	0.250	Scan Lens 2 Tilt (Y)
28	TETX	9	11	0.000	-0.250	0.250	Tube Lens Tilt (X)
29	TETY	9	11	0.000	-0.250	0.250	Tube Lens Tilt (Y)
30	TETX	12	19	0.000	-0.050	0.050	Objective Assembly Tilt (X)
31	TETY	12	19	0.000	-0.050	0.050	Objective Assembly Tilt (Y)
32	TIRR	3		0.000	-0.200	0.200	Default irregularity tolerances.
33	TIRR	4		0.000	-0.200	0.200	
34	TIRR	5		0.000	-0.200	0.200	
35	TIRR	6		0.000	-0.200	0.200	
36	TIRR	7		0.000	-0.200	0.200	
37	TIRR	8		0.000	-0.200	0.200	
38	TIRR	9		0.000	-0.200	0.200	
39	TIRR	10		0.000	-0.200	0.200	
40	TIRR	11		0.000	-0.200	0.200	
41	TIRR	12		0.000	-0.200	0.200	
42	TIRR	13		0.000	-0.200	0.200	
43	TIRR	14		0.000	-0.200	0.200	
44	TIRR	16		0.000	-0.200	0.200	

Supplementary Figure 3: Tolerancing analysis, from the scanner forward without objective displacements. The analysis was conducted by shifting positions and tilt on each lens assembly apart from the objective set. a) PSF of the center of the field (top panel), at a scan angle corresponding to half of the FOV (middle panel), and at the edge of the FOV (bottom panel). Overlaid lines show 50 monte carlo simulation runs of the tolerancing analysis. b) RMS wavefront error over 50 monte carlo simulations. c) Tolerance Editor for these results, note that objective parameters are excluded.

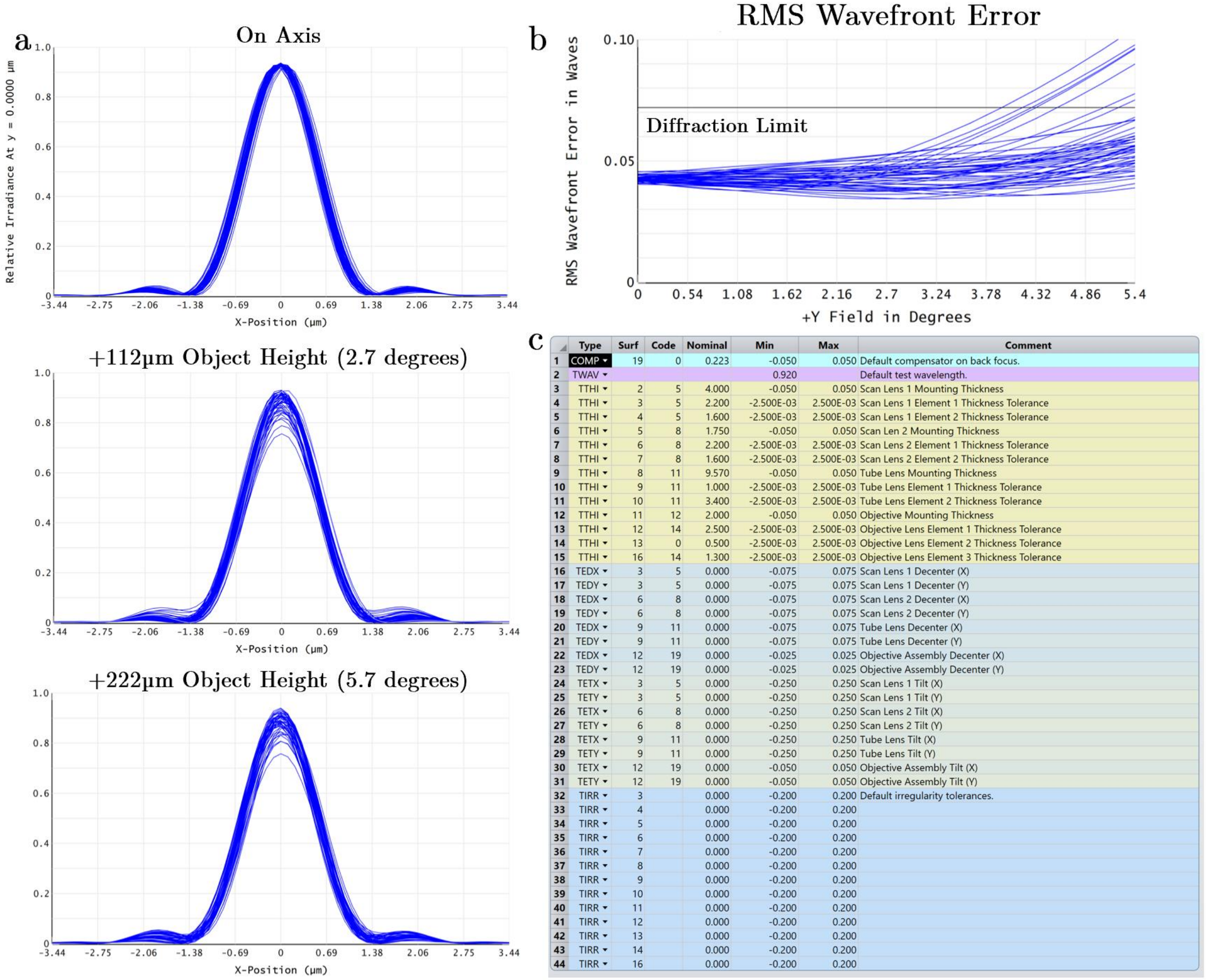

	Type	Surf	Code	Nominal	Min	Max	Comment
1	COMP	19	0	0.223	-0.050	0.050	Default compensator on back focus.
2	TWAV				0.920		Default test wavelength.
3	TTHI	2	5	4.000	-0.050	0.050	Scan Lens 1 Mounting Thickness
4	TTHI	3	5	2.200	-2.500E-03	2.500E-03	Scan Lens 1 Element 1 Thickness Tolerance
5	TTHI	4	5	1.600	-2.500E-03	2.500E-03	Scan Lens 1 Element 2 Thickness Tolerance
6	TTHI	5	8	1.750	-0.050	0.050	Scan Len 2 Mounting Thickness
7	TTHI	6	8	2.200	-2.500E-03	2.500E-03	Scan Lens 2 Element 1 Thickness Tolerance
8	TTHI	7	8	1.600	-2.500E-03	2.500E-03	Scan Lens 2 Element 2 Thickness Tolerance
9	TTHI	8	11	9.570	-0.050	0.050	Tube Lens Mounting Thickness
10	TTHI	9	11	1.000	-2.500E-03	2.500E-03	Tube Lens Element 1 Thickness Tolerance
11	TTHI	10	11	3.400	-2.500E-03	2.500E-03	Tube Lens Element 2 Thickness Tolerance
12	TTHI	11	12	2.000	-0.050	0.050	Objective Mounting Thickness
13	TTHI	12	14	2.500	-2.500E-03	2.500E-03	Objective Lens Element 1 Thickness Tolerance
14	TTHI	13	0	0.500	-2.500E-03	2.500E-03	Objective Lens Element 2 Thickness Tolerance
15	TTHI	16	14	1.300	-2.500E-03	2.500E-03	Objective Lens Element 3 Thickness Tolerance
16	TEDX	3	5	0.000	-0.075	0.075	Scan Lens 1 Decenter (X)
17	TEDY	3	5	0.000	-0.075	0.075	Scan Lens 1 Decenter (Y)
18	TEDX	6	8	0.000	-0.075	0.075	Scan Lens 2 Decenter (X)
19	TEDY	6	8	0.000	-0.075	0.075	Scan Lens 2 Decenter (Y)
20	TEDX	9	11	0.000	-0.075	0.075	Tube Lens Decenter (X)
21	TEDY	9	11	0.000	-0.075	0.075	Tube Lens Decenter (Y)
22	TEDX	12	19	0.000	-0.025	0.025	Objective Assembly Decenter (X)
23	TEDY	12	19	0.000	-0.025	0.025	Objective Assembly Decenter (Y)
24	TETX	3	5	0.000	-0.250	0.250	Scan Lens 1 Tilt (X)
25	TETY	3	5	0.000	-0.250	0.250	Scan Lens 1 Tilt (Y)
26	TETX	6	8	0.000	-0.250	0.250	Scan Lens 2 Tilt (X)
27	TETY	6	8	0.000	-0.250	0.250	Scan Lens 2 Tilt (Y)
28	TETX	9	11	0.000	-0.250	0.250	Tube Lens Tilt (X)
29	TETY	9	11	0.000	-0.250	0.250	Tube Lens Tilt (Y)
30	TETX	12	19	0.000	-0.050	0.050	Objective Assembly Tilt (X)
31	TETY	12	19	0.000	-0.050	0.050	Objective Assembly Tilt (Y)
32	TIRR	3		0.000	-0.200	0.200	Default irregularity tolerances.
33	TIRR	4		0.000	-0.200	0.200	
34	TIRR	5		0.000	-0.200	0.200	
35	TIRR	6		0.000	-0.200	0.200	
36	TIRR	7		0.000	-0.200	0.200	
37	TIRR	8		0.000	-0.200	0.200	
38	TIRR	9		0.000	-0.200	0.200	
39	TIRR	10		0.000	-0.200	0.200	
40	TIRR	11		0.000	-0.200	0.200	
41	TIRR	12		0.000	-0.200	0.200	
42	TIRR	13		0.000	-0.200	0.200	
43	TIRR	14		0.000	-0.200	0.200	
44	TIRR	16		0.000	-0.200	0.200	

Supplementary Figure 4: Tolerancing analysis from the scanner forward with objective displacements specifically to understand contributions from objective misalignment in combination with other optical tolerances. a) PSF of the center of the field (top panel), at a scan angle corresponding to half of the FOV (middle panel), and at the edge of the FOV (bottom panel). Overlaid lines show 50 monte carlo simulation runs of the tolerancing analysis. b) RMS wavefront error over 50 monte carlo simulations. c) Tolerance Editor for these results with objective displacement and tilt.

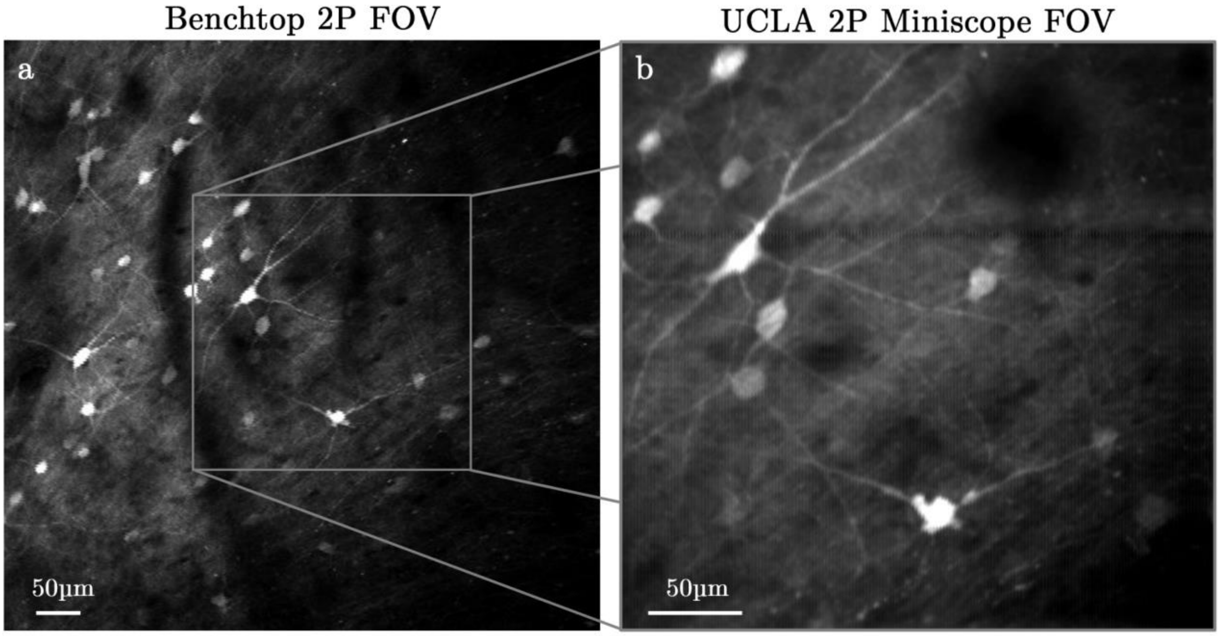

Supplementary Figure 5: Comparison of benchtop and UCLA 2P Miniscope fields of view *in-vivo*. a) Benchtop microscope with an excitation NA of 0.5 imaging GCaMP-expressing neurons in CA1. b) Same FOV measured with the UCLA 2P Miniscope resolving the same neurons and projections

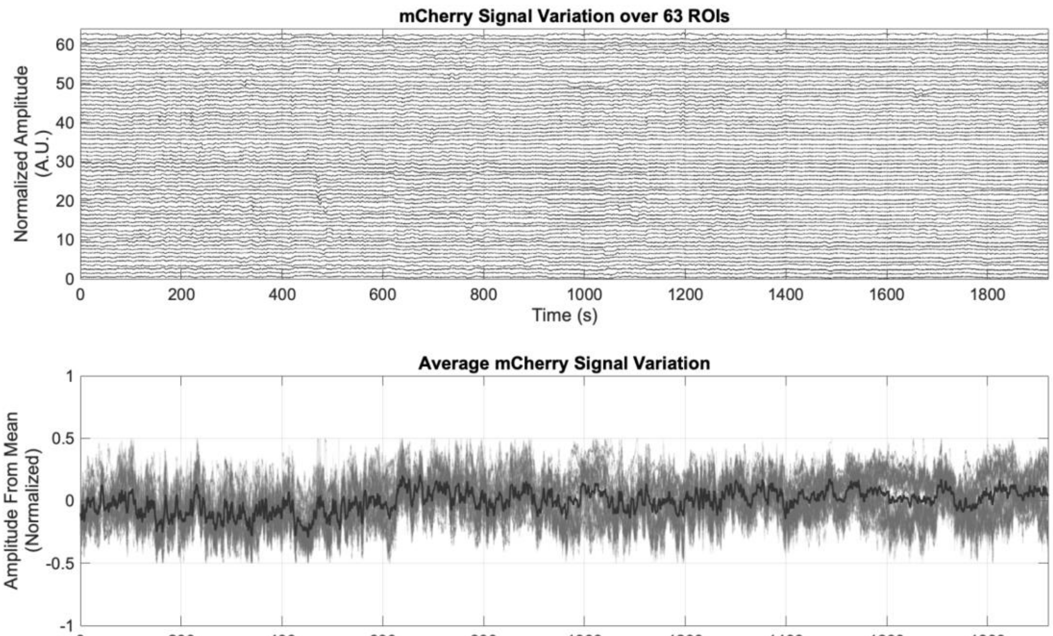

Supplementary Figure 6: Monitoring Stability of mCherry Fluorescence over 32 Minutes of Free Behavior. 63 mCherry-expressing ROIs were monitored for the duration of the full 32-minute free behavior RSC recording. Localized areas of mCherry expression were selected and monitored over the course of the recording after linear unmixing.

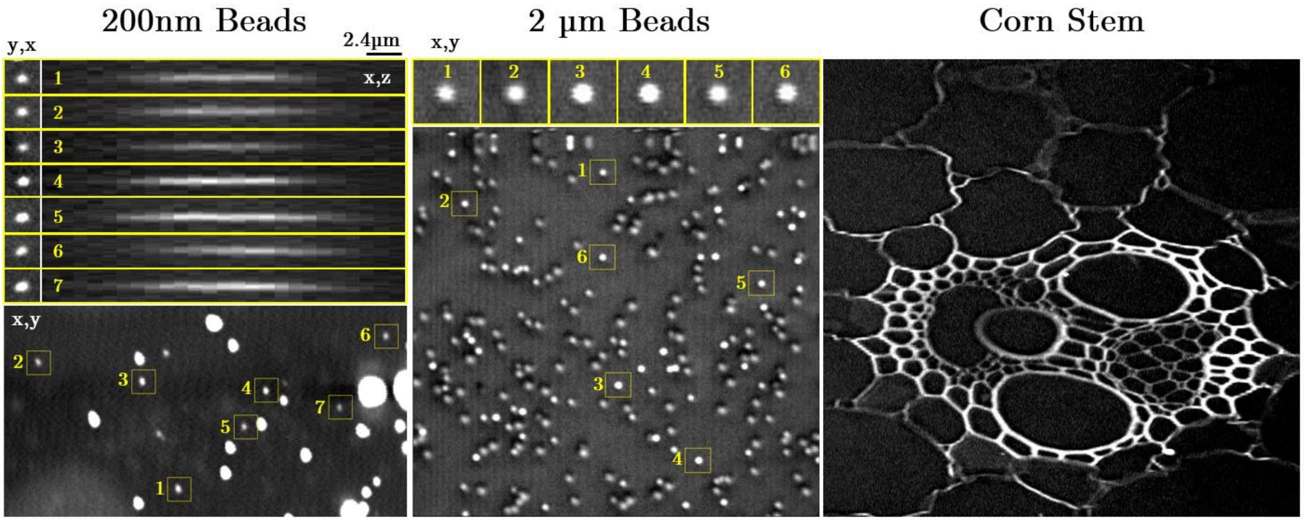

Supplementary Figure 7: Additional Calibration Images. Left panel shows individual 200nm beads as well as projections from single beads in the y,x and x,z planes. The images presented are mean projections across the 40um image volume, taking care to not analyze aggregates of microspheres. Bottom image in the FOV is a single frame near the focal plane. Middle panel shows 2um beads. The right panel shows an image of corn stem which was used to experimentally measure the FOV by translating one corner of a clear aspect of the sample from edge-to-edge with a calibrated motorized stage and recording the displacement.

Figure	Brain Region	Frame Rate	FOV Size (Approximate)	Resolution (Pixels)	Resonant Axis Drive Frequency
Figure 3, Panel D	CA1	8.62 Hz	~ 210um x 210um	512 x 354	1650Hz
Figure 4, Panel B	RSC	7.92 Hz	~ 285um x 285um	512 x 350	1650Hz
Figure 5, Panel C	DG	8.62 Hz	~ 206um x 206um	512 x 354	1650Hz
Supplemental Figure 2	DG	8.62 Hz	~ 206um x 206um	512 x 354	1650Hz
Supplemental Figure 5	CA1	8.62 Hz	~ 308um x 308um	512 x 354	1650Hz

Supplementary Table 1: Imaging Parameters for all datasets. Bidirectional scanning was used for all data collection. FOV size is reported after post-processing and motion correction.

Reference	Klioutchnikov, A. et al. Nat Methods (2020)	Klioutchnikov, A. et al. Nat Methods (2023)	Zhao, C. et al. Nat Methods (2023)	Zhao, C. et al. Opt Express (2023)	Zong, W. et al. Cell (2022)	Qian, L. et al. bioRxiv (2024)	Madruga, B.A. et al. bioRxiv (2024)
**Number of photons**	3p	3p	3p	2p	2p	2p	2p
**Emission NA, Excitation NA**	0.9, 0.48-0.54	0.9, 0.48/0.58	0.65, 0.55	0.45, 0.25	0.45-0.5, 0.45-0.5	0.95, 0.26	0.6, 0.36
**Lateral Resolution**	0.8 μm	1.18 / 1.11 μm	0.97-1.21 μm	1.47 μm	1.15-1.24 μm	0.97-0.99 μm	0.98 μm
**Axial Resolution**	5.3 μm	13.8 / 10.1 μm	7.21-8.51 μm	24.64 μm	12.8-17.8 μm	80.4-90.2 μm	10.18 μm
**FOV**	160 × 160 μm^2	300 × 300 / 200 × 200 μm^2	400 × 400 μm^2	1000 × 788 μm^2	420 × 420 / 500 × 500 μm^2	423 × 439 μm^2	445 x 380 μm^2
**Frame Rate**	27.78 Hz @ 120 × 120 pixels	10.6 Hz @ 273 × 280 pixels	15.93 / 8.35 Hz @ 128 × 128 / 200 × 200 pixels	9 Hz @ 600 × 512 pixels	40 / 15 Hz @ 256 × 256 pixels	9 Hz @ 512 × 512 pixels	8.62 Hz @ 512 x 354 pixels, 7.92 Hz @ 512 x 350 pixels
**Weight**	5g	2g	2.17g	2.5g	< 3g	2.6g	~4g

Supplementary Table 2: Comparison of key optical specifications from selected multiphoton miniature microscope systems in the literature. The following table was lightly modified from Qian, L. et al. bioRxiv (2024) and used with the consent of the corresponding author.
