# Supplementary material for: Open-source, high performance miniature 2-photon microscopy systems for freely behaving animals
Source: Nat Commun. 2025 Aug 3;16:7125. doi: 10.1038/s41467-025-62534-y (PMC12318034; doi:10.1038/s41467-025-62534-y)
Supplement: Supplementary file 6 — Reporting Summary [file 41467_2025_62534_MOESM6_ESM.pdf]

nature portfolio

Corresponding author(s): Peyman Golshani

Last updated by author(s): Jul 18, 2025

Reporting Summary

Nature Portfolio wishes to improve the reproducibility of the work that we publish. This form provides structure for consistency and transparency in reporting. For further information on Nature Portfolio policies, see our Editorial Policies and the Editorial Policy Checklist.

Statistics

For all statistical analyses, confirm that the following items are present in the figure legend, table legend, main text, or Methods section.

n/a	Confirmed	
☐	☒	The exact sample size (*n*) for each experimental group/condition, given as a discrete number and unit of measurement
☐	☒	A statement on whether measurements were taken from distinct samples or whether the same sample was measured repeatedly
☐	☒	The statistical test(s) used AND whether they are one- or two-sided<br>*Only common tests should be described solely by name; describe more complex techniques in the Methods section.*
☒	☐	A description of all covariates tested
☒	☐	A description of any assumptions or corrections, such as tests of normality and adjustment for multiple comparisons
☐	☒	A full description of the statistical parameters including central tendency (e.g. means) or other basic estimates (e.g. regression coefficient) AND variation (e.g. standard deviation) or associated estimates of uncertainty (e.g. confidence intervals)
☐	☒	For null hypothesis testing, the test statistic (e.g. *F*, *t*, *r*) with confidence intervals, effect sizes, degrees of freedom and *P* value noted<br>*Give P values as exact values whenever suitable.*
☒	☐	For Bayesian analysis, information on the choice of priors and Markov chain Monte Carlo settings
☒	☐	For hierarchical and complex designs, identification of the appropriate level for tests and full reporting of outcomes
☒	☐	Estimates of effect sizes (e.g. Cohen's *d*, Pearson's *r*), indicating how they were calculated

Our web collection on statistics for biologists contains articles on many of the points above.

Software and code

Policy information about availability of computer code

Data collection	The 2P miniature microscope primarily uses ScanImage (Basic Version) from MBF Bioscience to control the key aspects of the system. The electro-tunable lens was controlled using a custom-written MATLAB (2022b) script that runs alongside Scanimage during operation. Additionally, the software WaveForms from Digilent (v.3.20.1) was used to control some aspects of the microscope function along with ScanImage. Controlling high precision stages was accomplished with ThorLabs Kinesis Software (v. 1.14.30) to verify the PSF and FOV measurements. Behavioral video was captured using the native Windows camera application.
Data analysis	Calcium imaging data was analyzed using Suite2P (v.0.9.2) and behavioral videos were processed with DeepLabCut (v.2.3.8). Aligning behavioral and calcium imaging data was done manually with Microsoft Clipchamp (v.3.1.10620.0). Generating spatial firing maps were computed with custom MATLAB scripts based on past publications. FIJI/ImageJ was used to take cross-sections of fluorescent beads that were further analyzed and aligned in MATLAB with a custom script.

For manuscripts utilizing custom algorithms or software that are central to the research but not yet described in published literature, software must be made available to editors and reviewers. We strongly encourage code deposition in a community repository (e.g. GitHub). See the Nature Portfolio guidelines for submitting code & software for further information.

Data

Policy information about availability of data

All manuscripts must include a data availability statement. This statement should provide the following information, where applicable:

- Accession codes, unique identifiers, or web links for publicly available datasets
- A description of any restrictions on data availability
- For clinical datasets or third party data, please ensure that the statement adheres to our policy

Our manuscript details the development of an open-source miniature 2P microscope. Accordingly, we have placed all design files necessary for replication (mechanical, optical, electronic) on GitHub (https://github.com/golshanilab/UCLA_2P_Miniscope) along with the custom control software, the parameter files we used to analyze the presented data, and pre-trained deeplabcut models for behavioral analysis. Since the initial submission we have also added demo data and scripts to walk users through the entire process. Being open and making sure that others have access to the key data that is generated here (namely the design files) is critical and something we want to ensure is completed to the utmost extent.

Research involving human participants, their data, or biological material

Policy information about studies with human participants or human data. See also policy information about sex, gender (identity/presentation), and sexual orientation and race, ethnicity and racism.

Reporting on sex and gender	N/A
Reporting on race, ethnicity, or other socially relevant groupings	N/A
Population characteristics	N/A
Recruitment	N/A
Ethics oversight	N/A

Note that full information on the approval of the study protocol must also be provided in the manuscript.

Field-specific reporting

Please select the one below that is the best fit for your research. If you are not sure, read the appropriate sections before making your selection.

[x] Life sciences [ ] Behavioural & social sciences [ ] Ecological, evolutionary & environmental sciences

For a reference copy of the document with all sections, see nature.com/documents/nr-reporting-summary-flat.pdf

Life sciences study design

All studies must disclose on these points even when the disclosure is negative.

Sample size	We chose to select three key experimental sessions (in three freely behaving animals) as a proof of concept of the capacity of the imaging system in challenging biological conditions. This is to highlight the unique ability of the microscope relative to others in the literature.
Data exclusions	In practice, we have recorded far more than three imaging sessions with the microscope. We have chosen to only report datasets that represent clear use cases to demonstrate the additional experimental capacity the imaging system offers to researchers.
Replication	We have used the miniature microscope to resolve calcium dynamics across many animals over several experimental conditions to verify that these measurements can be repeatedly taken in demanding experimental conditions. There are not any specific biological findings that are in need of replication to our understanding.
Randomization	We only have an experimental group (meaning they received surgery and viral injection of calcium indicator) of animals as randomness is not a key aspect of the manuscript.
Blinding	Blinding was not relevant to our study as we were assessing optical performance of the microscope in vivo. There was no condition that required being blinded.

Reporting for specific materials, systems and methods

We require information from authors about some types of materials, experimental systems and methods used in many studies. Here, indicate whether each material, system or method listed is relevant to your study. If you are not sure if a list item applies to your research, read the appropriate section before selecting a response.

Materials & experimental systems

n/a	Involved in the study
☒	☐ Antibodies
☒	☐ Eukaryotic cell lines
☒	☐ Palaeontology and archaeology
☐	☒ Animals and other organisms
☒	☐ Clinical data
☒	☐ Dual use research of concern
☒	☐ Plants

Methods

n/a	Involved in the study
☒	☐ ChIP-seq
☒	☐ Flow cytometry
☒	☐ MRI-based neuroimaging

Animals and other research organisms

Policy information about studies involving animals; ARRIVE guidelines recommended for reporting animal research, and Sex and Gender in Research

Laboratory animals	We used adult (>P60) male and female mice (C57BL/6) for the imaging results in depicted in the manuscript.
Wild animals	This study did not involve the use of wild animals.
Reporting on sex	Recordings were completed in both male and female mice, but no sex differences were explicitly studied
Field-collected samples	The study did not include field-collected samples
Ethics oversight	The study was entirely conducted under NIH guidelines the UCLA Chancellor's Animal Research Committee (ARC) Protocol number: ARC-2006-066.

Note that full information on the approval of the study protocol must also be provided in the manuscript.

Plants

Seed stocks	N/A
Novel plant genotypes	N/A
Authentication	N/A
